# Antibacterial Properties of Graphene-Based Nanomaterials

**DOI:** 10.3390/nano9050737

**Published:** 2019-05-13

**Authors:** Parveen Kumar, Peipei Huo, Rongzhao Zhang, Bo Liu

**Affiliations:** 1 Laboratory of Functional Molecules and Materials, School of Physics and Optoelectronic Engineering, Shandong University of Technology, Xincun West Road 266, Zibo 255000, China; kumar@sdut.edu.cn (P.K.); peipeihuo@sdut.edu.cn (P.H.); 2 Analysis and Testing Center, Shandong University of Technology, Xincun West Road 266, Zibo 255000, China

**Keywords:** graphene, antibacterial, silver, antibiotics, photocatalytic

## Abstract

Bacteria mediated infections may cause various acute or chronic illnesses and antibiotic resistance in pathogenic bacteria has become a serious health problem around the world due to their excessive use or misuse. Replacement of existing antibacterial agents with a novel and efficient alternative is the immediate demand to alleviate this problem. Graphene-based materials have been exquisitely studied because of their remarkable bactericidal activity on a wide range of bacteria. Graphene-based materials provide advantages of easy preparation, renewable, unique catalytic properties, and exceptional physical properties such as a large specific surface area and mechanical strength. However, several queries related to the mechanism of action, significance of size and composition toward bacterial activity, toxicity criteria, and other issues are needed to be addressed. This review summarizes the recent efforts that have been made so far toward the development of graphene-based antibacterial materials to face current challenges to combat against the bacterial targets. This review describes the inherent antibacterial activity of graphene-family and recent advances that have been made on graphene-based antibacterial materials covering the functionalization with silver nanoparticles, other metal ions/oxides nanoparticles, polymers, antibiotics, and enzymes along with their multicomponent functionalization. Furthermore, the review describes the biosafety of the graphene-based antibacterial materials. It is hoped that this review will provide valuable current insight and excite new ideas for the further development of safe and efficient graphene-based antibacterial materials.

## 1. Introduction

The rapid increase in bacteria mediated infectious diseases has been one of the most potential threats to human health and the cause of distressing millions of people around the world [1]. However, antibacterial materials effectively protect human health from these deadly infectious diseases. However, abrupt misuse of traditional antibiotics have directed the path to evolve many drug-resistant bacteria, which leads to the irretrievable infections [2]. The antibacterial materials are generally assessed against the typical group of the pathogenic bacteria such as *Listeria monocytogenes*, *Staphylococcus aureus* (*S. aureus*), *Pseudomonas aeruginosa* (*P. aeruginosa*), *Salmonella typhimurium* (*S. typhimurium*), *Streptococcus mutans* (*S. mutans*), *Escherichia coli* (*E. coli*), *Staphylococcus epidermidis* (*S. epidermidis*) *Vibrio harveyi* (*V. harveyi*), and *Enterococcus faecalis* (*E. faecalis*), which are the main cause for several human infections and are serious threats to health [3,4]. Antibacterial materials such as metal ions/oxides [5], antibiotics [6], quaternary ammonium compounds [7], and antimicrobial peptides [8] have been used for the treatment of bacterial infections but suffering from several issues. Metal ions/oxides have a broad range of antimicrobial activities against fungal, viral, and bacterial pathogens, but they showed toxicity against some mammalian cells [9,10,11]. Antibiotics and quaternary ammonium salt can develop resistance after prolonged use [12], whereas the development of pure antimicrobial peptides as antibacterial agents have very high cost [13,14]. To conquer these problems, several non-traditional antibacterial materials such as metal nanoparticles (NPs) [15], carbon nanotubes [16], metal oxide NPs [17], and graphene-based materials [18] have been explored extensively. Among them, graphene-based materials present astonishing traits to combat bacterial infections.

Graphene, which is a two-dimensional (2D) nano-structure consisting of sp^2^ carbons, is a building block of several carbon allotropes including charcoal, graphite, bucky balls, and carbon nanotubes. Graphene emerged as a promising nanomaterial since its discovery in 2004 [19] because of its unique catalytic, optical, and electrical properties as well as exceptional physical properties such as a large specific surface area and mechanical strength [20,21,22,23,24]. In addition, graphene is renewable, cheap, and easier to obtain compared to other nanomaterials [25,26]. The antibacterial mechanism of graphene involves both physical and chemical modes of action. Physical damages are the most common and are induced by direct contact of the sharp edge of graphene with bacterial membranes. In addition, the photo-thermal ablation and wrapping are also involved in the mechanism associated with physical damage. The chemical modes of action are associated with oxidative stress generated by charge transfer and a reactive oxygen species (ROS). Hu et al. [25] in 2010 first reported the antibacterial activity of two graphene-based materials: graphene oxide (GO) and reduced graphene oxide (rGO) nanosheets, which significantly inhibited the *E. coli* bacterial growth. Inspired by this special property and enormous potential applications of graphene, scientists have devoted their efforts to explore the activity of graphene-based materials against the realm of bacterial cells. 

**Figure 1:**
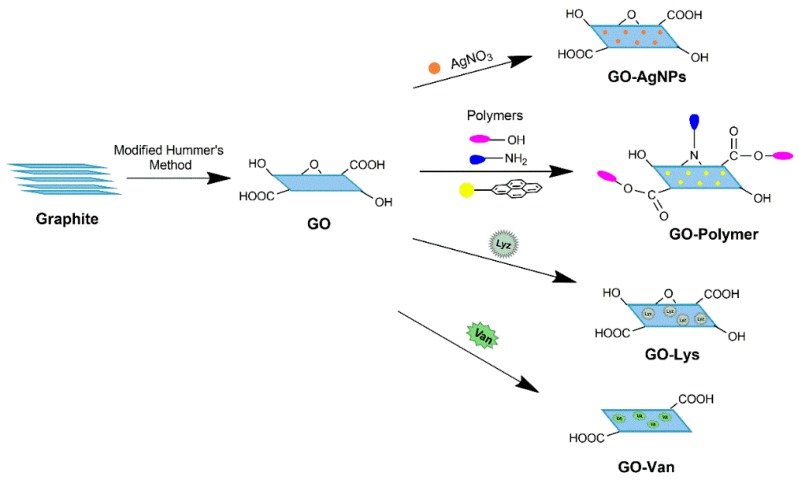
Schematic representation for the preparation of graphene-based nanocomposites.

So far, several routes for the production of graphene have been established and can be classified into two categories i.e., physical and chemical methods. Physical approaches involve the exfoliation of graphene layers from stacked graphite sheets via van der Waals force disruption using mechanical exfoliation [27,28] or direct liquid phase exfoliation [29,30]. Chemical approaches involve the preparation of various graphene containing materials via chemical reactions such as chemical vapor deposition (CVD) [31,32,33,34], epitaxial growth [35,36,37], chemical reduction of GO [38,39,40,41,42,43], and more. This review will enlighten the inherent antibacterial activity of the graphene-family and recent advances that have been made so far on graphene-based antibacterial materials covering the functionalization with silver (Ag) NPs, other metal ions/oxides/sulfides NPs, polymers, antibiotics, and enzymes along with their multicomponent functionalization (Figure 1). In addition, the biosafety of graphene-based antibacterial materials has also been discussed.

## 2. The Inherent Antibacterial Property of the Graphene-Family: Pristine Graphene, GO, and rGO

K. Novoselov and A. Geim first reported the graphene from the graphite by peeling their atomically thin layers using sticky tape [44]. Graphene is the strongest and thinnest material reported until now and exhibits remarkable conductance of electricity and heat, which arises from a thin single layer of carbon atoms orchestrated in a honeycomb pattern. Only a pristine form of graphene contains a complete sp^2^ hybridized single layer of carbon atoms with no defect. Various mechanisms of graphene regarding its antibacterial activity have been proposed such as oxidative stress, membrane stress, and electron transfer [45]. Graphene can physically damage the bacterial membranes by direct contact of its sharp edge. However, oxidative stress is the major cause of graphene toxicity [46] because the bacteria can no longer proliferate after deactivation of their proteins and lipids via ROS produced by the graphene. Moreover, graphene can also exhibit antibacterial activity by electron transfer since it can act as an electron acceptor and abstract electrons from bacterial membrane, which may compromise the membrane integrity [47]. Several recent studies have demonstrated that graphene exhibits promising antibacterial properties [48]. Tu et al. [49] showed that pristine graphene nanosheets induced degradation in the outer and inner membranes of *E. coli* and reduced their viability. Molecular dynamics simulations revealed that graphene nanosheets can insert into the cell membranes and extract phospholipids because of strong interactions between lipid molecules and graphene. This type of destructive extraction provided a novel mechanism of the antibacterial activity and cytotoxicity of graphene on the molecular basis. Li et al. [50] studied the antibacterial activity of monolayer graphene films on Cu, Ge, and SiO_2_ and revealed that graphene films on Ge and especially on Cu surprisingly inhibited the growth of *S. aureus* and *E. coli*. However, graphene films on SiO_2_ could not significantly restrict the proliferation of both bacteria. Pham et al. [51] found that pristine graphene nanosheets exhibited variable bactericidal efficiency towards *P. aeruginosa* and *S. aureus*. They studied the interactions between bacterial cell membranes and graphene surfaces and demonstrated that the density of the edges of graphene was the main parameter that contributed to the antibacterial property of the graphene films.

GO is the oxidized form of graphene having abundant oxygen bonds on its edges and defective sites, such as carboxylic (–COOH), carbonyl (–C=O), and hydroxyl (OH) groups on both accessible sides. The existence of these groups facilitates the interactions with biomolecules and induces bacterial death without an intracellular process. Antibacterial effect of GO is attributed to various mechanisms such as membrane stress, oxidative stress, entrapment, basal plane, and photo-thermal effect. Sharpe edges of the nanosheets of GO may physically damage the bacterial membranes and lead to the inactivation of bacteria due to leakage of the intracellular matrix [52,53,54,55]. Additionally, oxidative stress generated due to the production of ROS by GO can cause DNA damage and mitochondrial dysfunction, which results in bacterial inhibition [56,57]. The interaction between GO and the membrane of *E. coli* has been studied by Castrillón et al. [58] using specially prepared atomic force microscopy (AFM) probe coated with GO and found a repulsive interaction between them rather than an adhesive. In addition, bacteria get segregated from the environment and cannot proliferate due to blockage of gas/ion exchange. This is when it gets entrapped into the aggregated GO sheets [59]. Moreover, the GO nanosheets also show antibacterial activity dependence on lateral dimension against *E. coli*. Advincula et al. [60] showed that the antibacterial activity of flat GO films on poly(ethylene terephthalate) (PET) was surface-dependent and layer-dependent but not edge-dependent. The bactericidal efficacy of the films increased with the number of layers. In addition, GO may also be used for photo-thermal therapy since it shows the synergistic effect with laser energy and effectively enhances the antibacterial activity [61,62]. Hu et al. [25] reported the antibacterial property of GO nanosheets, which effectively inhibited the growth of *E. coli* with the viability loss of 98.5%. Liu et al. [54] evaluated the bactericidal activity of GO against *E. coli*, which displayed time-dependent and concentration-dependent behavior. Most of the bacteria inactivated in the first hour and the rate of cell death increased continuously with the concentration of GO. They have also evaluated the lateral dimension-dependent antibacterial property of well dispersed GO sheets against *E. coli* [63] in which larger GO sheets showed higher antibacterial activity than the smaller GO sheets and displayed different concentration-dependent and time-dependent behaviors. Larger GO sheets exhibited maximum cell viability loss after 1 h and showed the highest antibacterial activity at a relatively lower concentration. In comparison, smaller GO sheets displayed continuously increasing inactivation of *E. coli* up to 4 h and also an increasing trend in antibacterial activity with an increase in concentration. Chen et al. [64] investigated the antibacterial activity of GO against bacterial and fungal pathogens to understand the mechanism for its activity and revealed the powerful effect on all the tested pathogens. GO killed about 90% bacteria and represses about 80% macroconidia germination with the partial cell swelling and lysis at a concentration of 500 µg/mL and they have proposed the mechanism that GO intertwined the spores of fungi and bacteria resulting in the local perturbation of the cell membrane, which induced the electrolyte leakage of fungal spores and reduction of the bacterial membrane potential. They suggested that one of the major actions of the toxicity of GO against phytopathogens might be that GO interacted with pathogens by the mechanical wrapping and locally damaged the cell membranes and caused cell lysis.

rGO nanosheets are prepared by reducing the GO nanosheets with hydrazine [52], beta-mercaptoethanol [65], and dithiothreitol [66]. The rGO-based materials exhibit unique thermal, electronic, and mechanical properties and hold great promise in potential applications [67]. The antibacterial activity mechanisms of rGO depend mainly on oxidative stress and membrane stress. Several studies have been reported the bacterial toxicity of rGO-based materials and suggested that they could be used as efficient antimicrobial agents [54,65]. Gurunathan et al. [66] evaluated the antibacterial activity of synthesized rGO using cell viability, DNA fragmentation, and ROS production assays. rGO was synthesized by the reduction of GO with dithiothreitol and characterized with Raman spectroscopy and X-ray diffraction. The results revealed that rGO exhibited time-dependent and concentration-dependent antimicrobial activity against *E. coli*. Akhavan et al. [52] investigated the antibacterial activity of rGO and GO nanosheets in the form of nanowalls, which was deposited on the stainless steel against *E. coli* and *S. aureus* and revealed that *E. coli* were more resistant to cell membrane damage caused by nanowalls compared to *S. aureus*. Moreover, rGO nanowalls exhibited higher toxicity to bacteria than GO nanowalls. The reason for such a difference in activity between both nanowalls was suggested to be due to more sharp edges and better charge transfer of rGO nanowalls. Recently, Sengupta et al. [68] studied the influence of GO and rGO on *S. aureus* and *P. aeruginosa* and revealed that GO restricted the *S. aureus* and *P. aeruginosa* growth by 93.7% and 48.6%, respectively, whereas rGO restricted the growth by 67.7% and 93.3%, respectively. The reason for the antibacterial activity was attributed to GO destructing the bacteria by damaging the cell membrane through a chemical reaction whereas rGO induced the mechanical stress, which pierced the cell membrane. The shape and type of bacteria were the controlling factors for determining bactericidal efficacy of graphene.

## 3. Graphene-Based Composite Antibacterial Materials

Although the graphene family exhibits antibacterial properties, they tend to aggregate due to strong inter-plane interactions, which limit their surface area and modes of action. Therefore, to reduce the aggregation and enhance the antibacterial activity, various functionalization and surface modification with metal ions/oxides/sulfides NPs, polymers, antibiotics, and enzymes have been performed on graphene. Several metal ions/oxides/sulfides NPs such as AgNPs, titanium dioxide (TiO_2_) NPs, zinc oxide (ZnO) NPs, manganese disulfide (MnS_2_) NPs, and cadmium sulfide (CdS) NPs possess antibacterial properties with the generation of ROS via photocatalytic processes by irradiating the light on their surface with the energy greater than their band gap. Graphene is a good reservoir and acceptor of photo-generated electrons, which could efficiently promote photo-induced charge separation to enhance interfacial charge transfer and extend photo-generated electron/hole pairs life-time because of its remarkable electrical conductivity. Therefore, this markedly improves the photocatalytic activity of graphene-semiconductor nanocomposites. Furthermore, various polymers have recently been applied in various applications due to their antibacterial properties. In addition, graphene has also been used as a drug delivery system for various antibiotics and antibacterial enzymes. Consequently, many hybrid materials have been explored in recent years that combine the antibacterial properties of metal ions/oxides/sulfides NPs, polymers, antibiotics, and antibacterial enzymes with the inherent antibacterial property of graphene to achieve the enhanced effect. The bactericidal characters of graphene-based antibacterial materials are listed in Table 1 and summarized in the following sections.

### 3.1. Functionalization with Silver Nanoparticles

Ag and its compounds have been used since the age of ancient Egyptians and have been widely used in modern human medical science from the 20th century for their antibacterial activity [95,96]. The antibacterial and antiviral properties of Ag, Ag ions, and Ag-based compounds have been thoroughly investigated [97,98]. The Ag ions can penetrate the cells and destroy the membranes to inactivate bacteria [96]. AgNPs is a promising alternative to Ag salts and can exhibit the direct damage to bacterial cell membrane. Moreover, it can also generate ROS to fight against bacteria via photocatalytic activation using a light source. Several studies on antibacterial activity of AgNPs have been reported [99,100,101]. AgNPs may exhibit the combined antibacterial effect with the release of Ag ions. However, when bare AgNPs come in contact with bacteria, they aggregate and lose active surface area, which, thereby, show deceased antibacterial activity [102]. To conquer this, several nanocomposites composed of AgNPs with graphene have been prepared and studied against various bacterial strains [103,104,105,106,107,108,109,110,111,112,113,114,115] and showed promising antibacterial activities. These promising results drove the research toward producing more efficient GO/rGO-AgNPs based nanocomposites with improved antibacterial properties.

**Figure 2:**
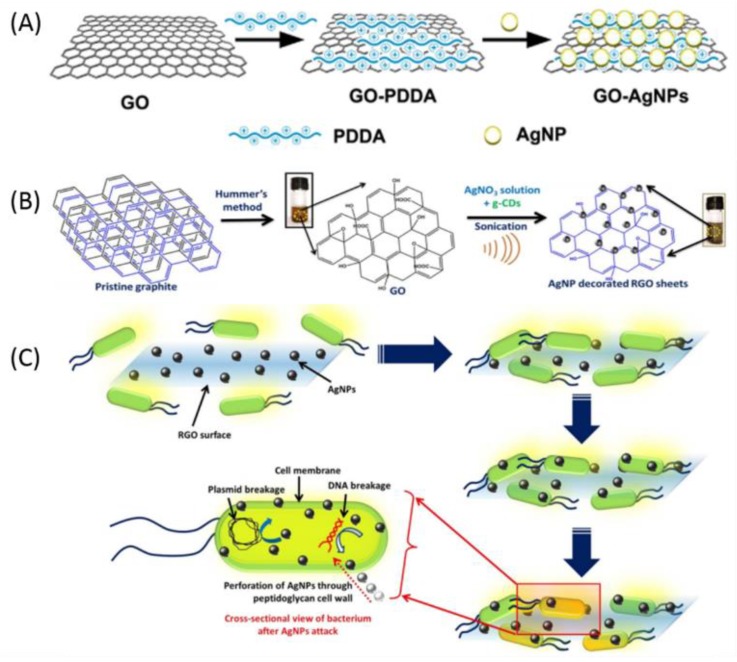
(**A**) Schematic representation of the self-assembly of PDDA and AgNPs onto GO nanosheets. Reproduced from Reference [116], with permission from Elsevier, 2013. (**B**) Synthesis of GO by hummer’s method followed by preparation of AgNP decorated nanosheets in situ by a sono-chemical method with gelatin derived C-dots. Reproduced from Reference [118], with permission from Elsevier, 2017. (**C**) Schematic representation of the ‘attack and kill’ mechanism performed by fabricated rGO-Ag nanocomposite. Reproduced from Reference [118], with permission from Elsevier, 2017.

Zhu et al. [116] used poly(diallyldimethylammonium chloride) (PDDA) as an adhesive agent for conjugating different densities, sizes, and forms of AgNPs to GO sheets via electrostatic interactions (Figure 2A). The synthesized GO-AgNPs composites showed enhanced colloidal stability, photo-stability, and antibacterial activities against *E. coli* and *B. subtilis* compared to AgNPs. Moreover, GO modified with smaller AgNPs showed higher antibacterial activity than GO modified with larger AgNPs. The functionalization of GO-AgNPs with polymers increases the antibacterial performance of the GO-AgNPs composites. Chen et al. [117] developed a method for synthesizing four different particle sizes (10, 30, 50, and 80 nm) of polyoxyethylene bis (amine) (PEG) directed AgNPs grown on the GO to form GO@PEG@AgNPs composites. The antibacterial activities against *E. coli* and *S. aureus* demonstrated that GO@PEG@AgNPs composites exhibited enhanced efficacy compared to AgNPs alone and the smallest composite (10 nm) showed higher antibacterial activity than other large composites (30, 50, and 80 nm).

Deng et al. [70] fabricated the visible light responsive GO-Ag_3_PO_4_ composites using an electrostatically driven self-assembly method by uniformly depositing Ag_3_PO_4_ particles on the surface of GO sheets. GO-Ag_3_PO_4_ composites exhibited enhanced bactericidal performance, which could be due to the more active adsorption sites on the surface of GO and reduced recombination of photo-generated electron-hole pairs because of excellent electronic conductivity and stored electricity of the GO. Most of the *E. coli* bacteria died within 30 min, while *S. aureus* bacteria almost completely died within 25 min under the visible light irradiation. Recently, Wang et al. [119] designed and fabricated the GO enwrapped AgCl/Ag nanocomposites (GO-AgCl/Ag) in which AgCl NPs were synthesized in the presence of GO sheets and AgNPs were coated on their surface by heat reduction. The prepared GO-AgCl/Ag nanocomposites exhibited improved stability and higher absorption properties in the visible light region. The antibacterial mechanism was investigated by evaluating the ROS level and membrane permeability and GO-AgCl/Ag nanocomposites exhibited enhanced antibacterial properties and bio-film disrupting ability. Most recently, Naeem et al. [120] used GO-Ag nanocomposites as an antibacterial agent for destroying the harmful micro-organisms in waste-water. AgNPs were integrated by the chemical reduction of Ag ions on the GO surfaces and the prepared GO-Ag nanocomposites exhibited remarkable performance in destroying harmful pathogens such as *E. coli* and *S. aureus*.

The use of rGO as an antibacterial agent is limited due to their tendency to aggregate because of strong van der Waals interactions among the sheets and this can be prevented by the formation of a nanocomposite by decorating metal ions/oxides NPs on its surfaces. The rGO-based nanocomposites provide unique properties such as large surface area and dispersibility in polar solvents such as water. Similar to GO, several rGO with AgNPs nanocomposites with improved antibacterial properties have been reported in recent years [69,121,122,123,124,125,126]. Moghayedi et al. [71] prepared and evaluated the antibacterial activity of rGO-AgNPs nanocomposites, which showed the time-dependent and concentration-dependent behavior against *E. coli*. Ganguly et al. [118] performed sonochemical green synthesis to prepare rGO decorated with AgNPs (Figure 2B) and confirmed the spherical shape of AgNPs over rGO sheets using UV-vis spectra and high-resolution TEM images. rGO-AgNPs nanocomposites showed the antibacterial effect via the ‘attack and kill’ mechanism by drastic breakage of the cell membrane and revealed significant antibacterial activity against *E. coli* (Figure 2C). Modification of rGO-AgNPs composites with L-cysteine further enhanced the antibacterial performance. Fathalipour et al. [127] prepared L-cysteine modified AgNPs decorated rGO nanocomposites in which L-cysteine played a triple role as a stabilizer, which reduces the agent and linker of AgNPs on the rGO surface. The prepared nanocomposites displayed enhanced electrocatalytic activity for the glucose as well as excellent antibacterial activity against *E. coli* and *S. aureus* tested by broth dilution methods and agar well diffusion. Huo et al. [72] fabricated the Ag/Ag_2_S/rGO nanocomposites using hydrothermal and UV-light assisted reduction methods, which showed outstanding antibacterial activity against *E. coli* (97.76%) with visible light irradiation in 24 h.

### 3.2. Photocatalytic Functionalization

Photocatalysis has recently appeared to be a green and effective method toward antimicrobial applications and proved to be efficient in the inactivation of various microorganisms by generating ROS. In photocatalysis, the light is used to initiate photons bombarding on the photocatalyst surface. Irradiation of light on the photocatalytic surface with the wavelength greater than the photocatalyst band gap excites electrons from the conduction band to the valance band. When the electron leaves valence band and absorbs onto the conduction band, it forms a positive hole in the valence band. In a conduction band, electrons react with O_2_ to form hydroperoxide radicals (HO_2_**^·^**) and superoxide radicals (O_2_**^−··^**). Simultaneously, generated positive holes in a valence band react with the H_2_O molecules to generate hydroxyl radicals (HO**^·^**). Several metal oxides/sulfides semiconductors with a narrow bandgap (CdS) and a wide bandgap (TiO_2_, ZnO, MnS_2_) act as a photo-catalyst to generate ROS to kill bacteria [128]. The photocatalytic activity of these semiconductors further enhances after coupling with graphene, which is attributed to remarkable electron conductivity of the graphene that provides a 2D network reservoir to accept as well as shuttle photogenerated electrons from the semiconductor, which results in the separation and prolonged lifetime of hole-electron pairs. This ultimately contributes to the enhancement of photocatalytic antibacterial activity. In addition, since the response edge of photo-catalysts is red-shift i.e., ultraviolet to visible region, graphene can also generate ROS while absorbing in an ultraviolet/visible region [129,130]. Photocatalytic functionalization of GO/rGO with various metal oxides/sulfides semiconductors (narrow and wide bandgap) has been proved to be highly effective to inactivate the bacteria by collective ROS generation to present their highly efficient photocatalytic antibacterial property.

Narrow bandgap semiconductors (e.g., CdS) can easily show photocatalytic activity in the visible light region and they have also been combined with graphene to form the nanocomposites for the photocatalytic antibacterial property. The CdS has an adequate bandgap (2.4 eV) to take a complete benefit of visible light (below 520 nm) and, moreover, it is a better material for CO_2_ and H_2_O reduction due to a more negative conduction band potential compared with many other metal oxide photocatalysts [131,132]. In addition, its band structure and positions can also be tuned by doping to significantly enhance the oxidation/reduction power and/or light-harvesting capacity, which results in magnified photoactivity. CdS/graphene composites have received great attention as a valuable photocatalyst in material and chemical science because of their remarkable photostability and photocatalytic activity under visible light irradiation. It is thermodynamically easier for photogenerated electrons to transfer from CdS to graphene. Moreover, intimate contact interface between CdS and graphene as well as remarkable capacity of graphene to store, capture, and shuttle the electrons further favor space separation of the charge carriers, which offers more chances to participate in the photocatalytic processes. Thus, graphene greatly elevates lifetime and availability of the charge carriers, which, thereby, enhances the CdS photocatalytic performance. Therefore, CdS is an attractive candidate for visible light mediated photo-catalysis and shows enhanced photocatalytic antibacterial activity by forming a nanocomposite with graphene [133]. Gao et al. [76] prepared the GO-CdS composite via two-phase mixing method in which CdS NPs were uniformly self-assembled onto the GO sheets at the toluene/water interface. GO-CdS composites exhibited enhanced photo-degradation of several water pollutants under visible light irradiation compared to pure CdS NPs. Moreover, 38.6 wt % of Cd^2+^ was leached out from CdS NPs aqueous solution while only 3.5 wt % of Cd^2+^ was leached out from GO-CdS composites because of the interaction between CdS NPs and GO sheets, which prevented the leaching of Cd^2+^ by inhibiting the photo-corrosion of CdS. Approximately 100% of *E. coli* and *B. subtilis* died under visible light irradiation within 25 min. Such high activity of GO-CdS composites could be attributed to the reduction in a recombination rate of generated hole-electron pairs due to effective charge transfer from CdS to GO sheets as well as the elimination of aggregation of CdS NPs due to their uniform deposition of the GO sheets. Deng et al. [134] prepared the GO-CdS nanocomposites using a two-step solvothermal process and exhibited higher inactivation efficiency toward *E. coli* under visible light irradiation than pure CdS NPs. However, GO-CdS nanocomposites showed a decrease in toxicity in the presence of humic acid, which might be due to the fact that humic acid prevented the physical contact between GO-CdS nanocomposites and bacteria cells as well as an antioxidant to react with the generated ROS, which reduced the toxicity. Although narrow bandgap semiconductors show photocatalytic activity in a visible light region and, even after coupling with graphene, their photocatalytic activity was restricted to a visible light region.

However, wide bandgap semiconductors generally show photocatalytic activity in an ultraviolet region. The photosensitization process of wide bandgap semiconductors can be transformed to exhibit visible light photo-activity after coupling with graphene [135,136]. TiO_2_ (bandgap, 3.2 eV) is widely used because of its high catalytic activity, low toxicity, excellent chemical/thermal stability, and low cost [137,138]. Several studies in recent years have shown the powerful photocatalytic antibacterial properties of GO-TiO_2_ composites [139,140,141,142]. Chang et al. [143] prepared magnetic GO-TiO_2_ (MGO-TiO_2_) composites and investigated the antibacterial activity against *E. coli*. The MGO-TiO_2_ composites almost completely inactivated the *E. coli* under solar irradiation within 30 min with the most effective concentration of 180 mg/L. Stan et al. [144] coated the cotton fabrics with rGO-TiO_2_ nanocomposites in order to gain new features in the fabrics such as self-cleaning, mechanical strength, and antimicrobial properties. The cotton fabrics were treated with rGO decorated TiO_2_ NPs co-doped with 1% iron and nitrogen atoms. The rGO-TiO_2_ nanocomposites coating on cotton fabrics were found to be harmless on human skin cells (CCD-1070Sk) and significantly inhibited the growth of *E. faecalis* and *S. aureus*. These findings confirmed the potential of rGO-TiO_2_ nanocomposite modified fabrics for developing novel antimicrobial and self-cleaning fabrics. Raja et al. [73] reported the hydrothermal preparation of the rGO-TiO_2_ nanocomposites and their efficiency in decomposing the ciprofloxacin under the visible light irradiation. rGO-TiO_2_ nanocomposites exhibited higher and faster degradation of ciprofloxacin compared to pure TiO_2_ and commercial TiO_2_-P25 under visible light irradiation, which was supposed to be due to their visible light activity and reduced hole-electron recombination. Antibacterial activity studies of rGO-TiO_2_ nanocomposites revealed higher activity against *S. aureus* compared to *E. coli*. Guo et al. [145] used rGO-TiO_2_ nanocomposites to improve the catalytic performance and also applied for the synergetic degradation of fluoroquinolone (flumequine) in the pulse discharge plasma (PDP) system. Light absorption range could be extended to the visible light and recombination rate of hole-electron pairs was declined in rGO-TiO_2_. The highest flumequine removal efficiency of 99.4% was achieved in PDP/rGO-TiO_2_ system at 5% rGO content, which was 34.6% and 23.7% higher than for sole PDP and PDP/TiO_2_ systems, respectively. Moreover, rGO-TiO_2_ nanocomposites decomposed the O_3_ and improved the generation of H_2_O_2_ and HO**^·^**. The toxicity of flumequine was evaluated according to the inhibition level of photo-bacterium Vibrio fischeri (*V. fischeri*) and found significant reduction in toxicity of the rGO-TiO_2_ treated flumequine solution after 60 min. 

**Figure 3:**
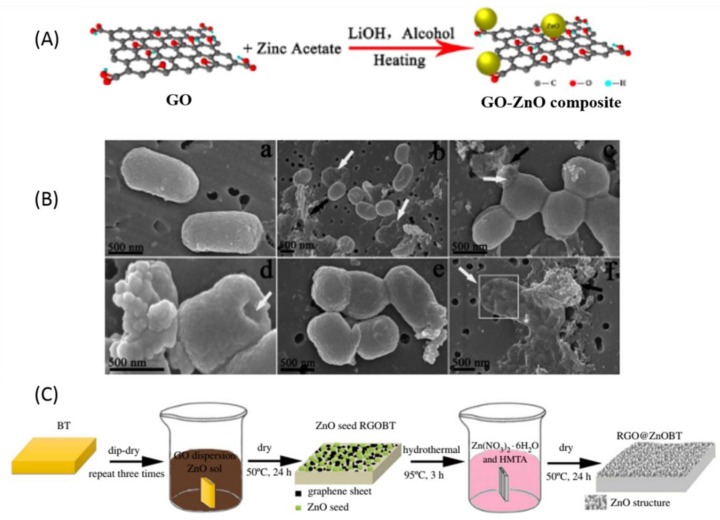
(**A**) Synthetic procedure for GO-ZnO composites from GO nanosheets. Reproduced from Reference [152], with permission from American Chemical Society, 2014. (**B**) SEM images of *E. coli*: control (a) treated with GO-ZnO-1 for 24 h (b–d), and treated with GO-ZnO-2 for 24 h (e,f). Reproduced from Reference [152], with permission from American Chemical Society, 2014. White square: cytoplasm leakage. Black arrows: GO-ZnO composites. White arrows: broken *E. coli*. (**C**) Schematic illustration of the synthetic procedure of RGO@ZnOBT [157].

ZnO (bandgap 3.3 eV) is one of the most interesting semiconductor photocatalysts widely used as an antibacterial agent [146]. However, it can only be activated under UV irradiation because of a wide bandgap and it also exhibits reduced photo-catalytic efficiency due to a high recombination rate of the photo-generated holes/electrons [147,148]. To conquer these shortcomings, several studies on the preparation of GO/rGO-ZnO composites with improved antibacterial activity have been reported [74,75,149,150,151]. Wang et al. [152] prepared GO-ZnO composites by a facile one-pot reaction to achieve superior antibacterial property with a very low effect on the HeLa cell viability in different concentrations, which is attributed to the synergetic effect of ZnO NPs and GO (Figure 3A,B). Wu et al. [153] investigated the mechanism of visible-light driven photocatalytic inactivation of *E. coli* K-12 bacteria by GO-ZnO composites and found that GO facilitated the charge transfer, which boosted the bulk production of ROS such as HO**^·^**, which was identified from the conduction band of ZnO and provided the remarkable enhancement in the inactivation efficiency. Chung et al. [154] fabricated the polysulfone-nanohybrid membranes with GO-ZnO composites via a wet phase inversion technique for the enhanced antibacterial and antifouling properties. The membranes were embedded with ZnO (1, 2, and 3 wt %) and GO-ZnO (0.1, 0.3, and 0.6 wt %). However, the membrane embedded with 2 wt % ZnO and 0.6 wt % GO-ZnO showed significantly enhanced properties such as permeability, hydrophilicity, antifouling, and antibacterial activity. Liu et al. [155] developed the ZnO/graphene quantum dot (ZnO-GQD) nanocomposites via the hydrothermal method and evaluated the antibacterial activity on *E. coli* by a standard plate count method. Markedly enhanced antibacterial activity was found for ZnO-GQD nanocomposites under UV photo-irradiation compared to ZnO and GQD separately, which was ascribed to enhanced ROS production under the UV photo-irradiation with minor contributions from the damage of the membranes. Zhong et al. [156] investigated the antibacterial activity of ZnO NPs decorated spindle-shaped GO and revealed that the nanocomposites could prevent the proliferation of gram-negative (*E. coli* and *S. typhimurium*) and gram-positive (*B. subtilis* and *E. faecalis*) bacteria by destroying the bacterial membrane due to the release of the Zn^2+^ ion and the generation of ROS. Minimum inhibit concentration (MIC) values of the nanocomposites for gram-positive bacteria and gram-negative bacteria were about 31.25 and 15.62 µg/mL, respectively. Bamboo is vulnerable to mold and attack by various fungi due to its high content of sugar and starch. Therefore, to prepare bacterial resistant bamboo-based outdoor materials, Wang et al. [157] coated the bamboo with GO-ZnO nanocomposites prepared via dip-dry and hydrothermal process and found significantly enhanced antibacterial activity on *Penicillium citrinum*, *Trichoderma viride*, *Aspergillus niger*, and *E. coli* for the treated bamboo compared to the untreated one. The inhibition zone width of the original bamboo timber (BT) samples was 0 mm, whereas the inhibition zone width of the RGO@ZnOBT samples was 3 mm (Figure 3C). Vanitha et al. [158] fabricated the Ce doped ZnO-rGO nanocomposites by a one-pot hydrothermal process without surfactant and characterized its size, morphology, and crystallography using various spectroscopic techniques. The doping of Ce into ZnO-rGO nanocomposites improved the antibacterial activity toward *V. harveyi* and *B. subtills*.

Metal sulfides have been evaluated as an excellent standalone photocatalyst or as a component of photocatalyst composite due to its attractive merits such as large specific surface area, electrochemical-electrical properties, environmental stability, larger abundance, and low cost [159]. Graphene provides a valuable combination with MnS_2_ (bandgap, ~3.7 eV) to form effective antibacterial material because, along with pristine graphene mechanism activity, it can also stabilize the catalyst and offer a 2D plan for their deposition [160]. Fakhri et al. [161] analyzed the photo-catalytic and antibacterial activity of rGO-MnS_2_ composites and revealed that rGO-MnS_2_ composites showed good photo-catalytic activity and were able to inhibit the growth of *E. coli*. The width of the MnS_2_ NPs and rGO-MnS_2_ composites were found to be around 20 and 5 nm, respectively, and MIC and minimum bactericidal concentration (MBC) values for rGO-MnS_2_ composites were 4.0 and 32.0 µg/mL.

### 3.3. Functionalization with Other Metals and Metal Oxides 

In recent years, several other metals and metal oxides (Cu, W, Fe, and Co) were applied to form a hybrid with GO/rGO in order to enhance synergistic antibacterial properties of the resultant composites. The obtained hybrids were evaluated against several bacterial pathogens and resulted in higher antibacterial activity than GO/rGO [78,79,80,81,162]. Metal oxides can kill the bacteria by means of releasing metal ions and generating ROS when in contact with bacteria [163]. Therefore, the antibacterial activity of metal oxides is associated with the release of metal ions and morphology such as shape and size. Small cubic Cu_2_O crystals with maximum exposed (100) crystal plane exhibits the best antibacterial performance because ROS largely produce on a (100) crystal plane [164]. Cu_2_O is more likely to release the Cu ions due to a large specific surface area to kill the bacteria. It is markedly promising to explore graphene nanosheets as support material for various metal oxide NPs to fabricate hybrid nanocomposites due to their unique 2D nanostructure and remarkable properties. Zavareh et al. [165] prepared the Cu^2+^ bound GO and evaluated the antibacterial activity against various bacterial strains. The Cu^2+^ modified GO exhibited high antibacterial activity and high capacity for aniline adsorption whereas unmodified GO showed almost no adsorption. Therefore, Cu^2+^ modified GO could be used as drinking water disinfection filters in water treatment plants. Deng et al. [166] prepared the Cu NPs decorated graphene sponge (GS-Cu) as a bactericidal filter for the inactivation of *E. coli*. GS-Cu consisted of well dispersed Cu NPs on the 3D porous graphene network and showed high antibacterial efficiency against *E. coli* induced by membrane damage. In addition, the Cu ions released from the GS-Cu was below a drinking water standard. Yang et al. [77] prepared the rGO-cuprous oxide (rGO-Cu_2_O) nanocomposites by reducing copper sulfate supported on the GO with ascorbic acid in the presence of PEG and NaOH. rGO promoted the separation of charge carriers of Cu_2_O NPs to increase oxidative stress and protect the Cu_2_O NPs from falling in PBS solution to prolong the production of ROS. The synergistic effect of elevated ROS generation ability, uniform dispersion of rGO-Cu_2_O nanocomposites, and sustained release of Cu ions resulted in good antibacterial activity of around 70% and 65% toward *E. coli* and *S. aureus*, respectively.

Jeevitha et al. [167] prepared GO-WO_3_ nanocomposites to evaluate the antibacterial, photo-catalyst, and anticancer activity. Enhanced antibacterial activity was observed under sunlight compared to WO_3_ NPs, which could be attributed to an improved hole-electron pair separation rate. The anticancer activity of GO-WO_3_ nanocomposites was investigated on the A-549 cell line and IC_50_ was found to be 139.6 ± 4.53 μg/mL. Recently, Ahmed et al. [168] developed 2D rectangular sheets of WO_3_ and WO_3_ grown over rGO sheets (rGO-WO_3_) using the hydrothermal method. The antibacterial activity of WO_3_ sheets and rGO-WO_3_ composites was investigated on *B. subtilis* and *P. aeruginosa* for the first time and revealed that rGO-WO_3_ composites showed better activity than WO_3_ sheets and higher antibacterial effect was observed for *B. subtilis* compared to *P. aeruginosa*. Mahmoodabadi et al. [169] prepared GO-Fe_3_O_4_ nanocomposites using the solvothermal method with different weight ratios of GO and Fe_3_O_4_ such as 1:1, 1:3, and 1:5. The antibacterial properties of the prepared nanocomposites were investigated against *S. aureus* and *E. coli* using the MIC method and the colony counting method and revealed that GO-Fe_3_O_4_ nanocomposites with 1:5 wt % ratio exhibited lower toxicity and higher antibacterial activity compared to Fe_3_O_4_ NPs and GO sheets. Most recently, Sadhukhan et al. [170] reported the green and efficient synthesis of CdO decorated rGO nanocomposites (rGO-CdO) using one step co-precipitation and the hydrothermal method. The prepared nanocomposites were characterized by various spectroscopic techniques. Enhanced electrical conductivity and antibacterial activity were observed for rGO-CdO nanocomposites compared to CdO NPs and rGO. Therefore, these nanocomposites could be a safe and effective replacement for toxic CdO in various biological applications. Zhu et al. [171] used fungus hyphae as a templet for the assembly of Fe_3_O_4_ NPs and GO sheets to form the fungal hyphae/Fe_3_O_4_/GO (FFGS) composites by successively culturing fungus hyphae in the GO and Fe_3_O_4_ containing mediums. Fungal template enhanced the stability and dispersity of Fe_3_O_4_ and GO sheets. The FFGS composites showed much better adsorption of methyl violet and uranium compared to fungus hyphae and its hybrid with GO or Fe_3_O_4_, which might be due to its low zeta potential. Moreover, FFGS composites exhibited several other properties such as a simple synthetic procedure, easy magnetic separation, and excellent regeneration performance. Singh et al. [172] synthesized the GO via oxidation of graphite using a strong oxidizing agent to get -OH, -CO, epoxide, and -COOH functionalities on the surfaces and the edges of the GO sheets. The synthesized GO and Fe_3_O_4_ was used to prepare the GO-Fe_3_O_4_ nanocomposites and investigated its antibacterial activity against various bacterial strains such as *Klebsiella pneumoniae*, *Proteus hauseri*, *S. aureus,* and *Streptococcus pyogenes*. GO-Fe_3_O_4_ nanocomposites exhibited the effective bactericidal potential against all tested bacteria. Arshad et al. [173] studied the solar light activated GO-Fe_3_O_4_ nanocomposites for the antibacterial property. Magnetically separable GO-Fe_3_O_4_ nanocomposites inhibited the growth of *S. aureus* and *P. aeruginosa* 95.33% and 89.02%, respectively, under solar light irradiation.

### 3.4. Functionalization with Polymers

The graphene has poor solubility because of its tendency to aggregate due to strong inter-planer interactions, which severely limit its antibacterial application and this limitation can be conquered by incorporating the polymer matrix into the graphene to form stable graphene-polymer dispersion and avoid aggregation [174]. Polymer-based composites have received significant attention because of their tunable properties to develop high-performance nanocomposites for biomedical applications [175]. There are three main strategies to develop GO-polymer nanocomposites: (a) physical mixing of GO with the polymer, (b) covalent bonding of the polymer matrix via GO functional groups, and (c) attachment of GO with the aromatic group of the polymer via non-covalent interactions such as van der Waals forces, hydrophobic interactions, and π-π stacking. Recently, several polymer-graphene nanocomposites have been prepared to enhance antibacterial activity using polymer matrixes such as poly (vinyl alcohol) (PVA) [176,177], CS [178,179,180,181], poly (lactic acid) (PLLA) [182], poly-*N*-vinyl carbazole (PVK) [174,183,184,185], and poly-L-lysine (PLL) [186]. 

CS is a natural polysaccharide with interesting properties such as high biodegradability and a non-toxic nature, which attracted attention for various biomedical applications due to its excellent antimicrobial activity. Mazaheri et al. [180] prepared GO-CS composite layers using GO sheets with ~1 nm thickness and ~1 µm lateral dimensions. The elastic modulus and strength of the composite layers grew when increasing the GO content. The GO-CS composite layers exhibited significant antibacterial activity against *S. aureus*. Surface density of actin cytoskeleton fibers of the human mesenchymal stem cells cultured on CS and GO-CS composite (GO content 1.5 wt %) layers was nearly same, whereas it significantly decreased on increasing GO content of the composite to 3 and 6 wt %. Sundar et al. [84] also synthesized GO-CS nanocomposites and characterized them using several spectroscopic techniques for the evaluation of the antibacterial property. UV-visible spectroscopy and FTIR confirmed the formation of GO-CS nanocomposites. The antibacterial activity was tested against *E. coli* and *B. subtillis* and results showed that GO-CS (2 wt % GO content) nanocomposites exhibited higher antibacterial activity compared to individual activity of GO sheets or CS against both the bacteria. Recently, Grande et al. [187] fabricated cross-linked GO-CS nanocomposite films for potential application in food packaging. The nanocomposite films were prepared by cross-linking CS with GO at 120 °C and showed good mechanical strength and thermal stability. GO-CS nanocomposite films (0.6 wt % GO content) exhibited 54.93% and 22.83% microbial inactivation against *B. subtillis* and *E. coli*, respectively. The cross-linking of GO with CS significantly enhanced the mechanical strength, thermal stability, and antimicrobial activity of the films, which made them a remarkable candidate for application in food packaging.

**Figure 4:**
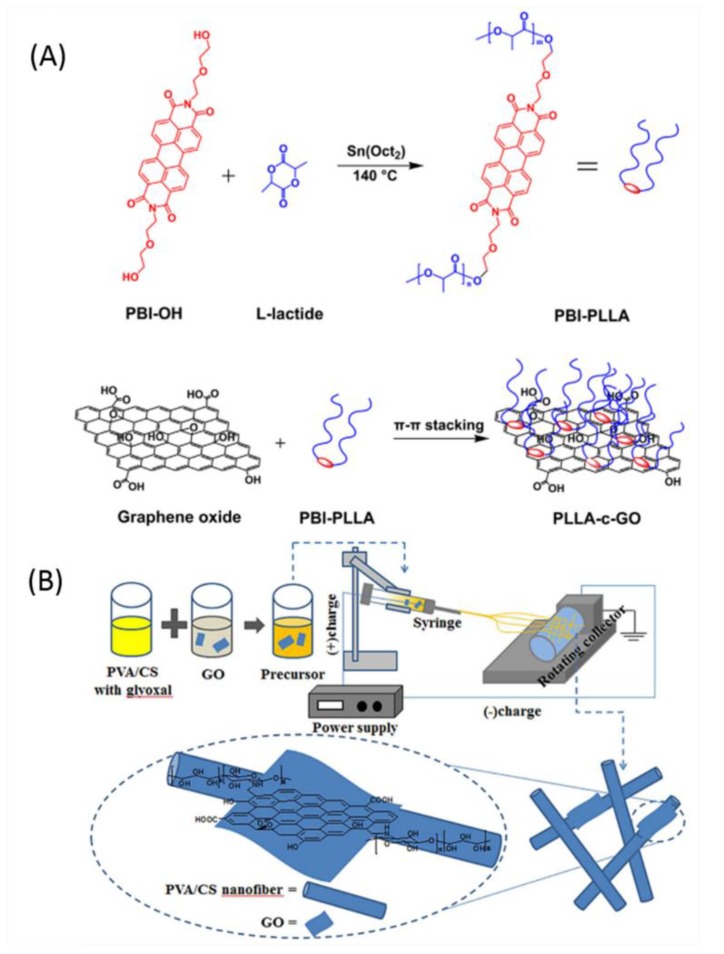
(**A**) Schematic illustration of the preparation of PLLA-c-GO from PLLA and GO. Reproduced from Reference [188], with permission from Elsevier, 2017. (**B**) Procedure for the fabrication of PVA/CS nanofibers with self-assembled GO nanosheets. Reproduced from Reference [189], with permission from Elsevier, 2014.

PLLA is one of the most promising biodegradable polymers that has been widely used for biomedical applications due to its appropriate biocompatibility, renewability, and mechanical properties. Yang et al. [188] prepared GO-PLLA films by the simple solution casting method using commercial PLLA. Perylene bisimides-containing PLLA was conjugated to GO by π-π stacking to form the PLLA-conjugated GO (PLLA-c-GO) (Figure 4A) and PLLA-GO films were prepared by solution casting of PLLA-c-GO and commercial PLLA dissolved in chloroform. The PLLA-GO films showed enhanced thermal stability and flexibility. The PLLA-GO films resulted in significant antibacterial activity against *E. coli*, *S. aureus*, and *B. subtilis* with an 80% bacterial colony number reduction as well as low toxicity against mammalian cells, such as macrophage and L929 cells. 

The guanidine polymer has a wide spectrum antimicrobial activity, good biocompatibility, and excellent biocide efficiency. However, because it is water soluble, it is strenuous to recycle and, as a result, can cause secondary contaminations [190] and its use as additives for the industrial goods may lead in impaired antibacterial fastness of final products [191]. Therefore, guanidine polymer could be combined with graphene to form the hybrid composite materials due to excellent antibacterial properties. Li et al. [85] reported the polyhexamethylene guanidine hydrochloride (PHGC) and polyethylene glycol (PEG) functionalized GO (GO-PEG-PHGC) composites synthesized via the modified Hummer’s method and covalently conjugated PHGC and PEG to test their antibacterial activity against *E. coli* and *S. aureus*. GO-PEG-PHGC composites exhibited higher antibacterial activity compared to individual GO, PEG-GO, or PHGC-GO against the bacteria, which might be attributed to the better dispersion of GO-PEG-PHGC composites in the presence of PEG. This resulted in greater contact with the bacterial membrane to cause greater damage.

Recently, in view of the toxicity of antibacterial agents to human cells, Liu et al. [192] modified the silicon rubber surface with GO coating and investigated the antibacterial activity against *E. coli* and *S. aureus*. The flat and featureless coating of GO powder on the silicon rubber surface showed strong antibacterial activity toward *E. coli* than *S. aureus* and the oxidative stress was considered to be the main cause of the antibacterial activity. This biocompatible, safe, and easy to recycle GO coating on silicon rubber provides an excellent alternative for application in the antibacterial instrument such as catheters, implants, and so on. Most recently, Tu et al. [83] prepared the poly(dimethylsiloxane) (PDMS) modified dopamine methacrylamide (DMA)-GO (PDMS-GO-DMA) composites and characterized with various spectroscopic techniques. The PDMS-GO-DMA composites exhibited good antibacterial activity against *E. coli* and *S. aureus* compared to the native PDMS surface, as determined using visible spectrophotometry and classic colony methods. Furthermore, PDMS-GO-DMA composites exhibited excellent biocompatibility on HepG2 and A549 cell lines. Thus, these findings provide their possible use as antibacterial functional surfaces in the biomedical micro-device applications.

Electrospinning is a versatile technique to prepare the fiber materials including polymer fibers, composite fibers, porous structure fibers, and more [193,194]. Electrospun nonwoven fibrous membranes fabricated with biocompatible polymers have been extensively used for biomedical applications, such as artificial tissue scaffold, wound dressing, and so on [195]. By the assistance of electrospinning technology, Liu et al. [189] fabricated the poly(vinyl alcohol) (PVA)/CS/GO composite (PVA-CS-GO) nanofibers and found that the average diameters of PVA-CS-GO composite nanofibers decreased when increasing the GO contents (Figure 4B). The addition of GO sheets to PVA/CS nanofibers increased the bacterial inhibition, which can serve as a promising candidate in wound healing, tissue engineering, and drug delivery systems. Recently, Yang et al. [82] also prepared PVA-CS-GO composite nanofibers via electrospinning and exhibited good antibacterial activity against *E. coli* and *S. aureus*. Most recently, Park et al. [196] also used the electrospinning technique to prepare the composite nanofibers for super-hydrophilic and antibacterial properties. The quaternary ammonium-functionalized amphiphilic di-block copolymers consisting of a hydrophobic poly(methyl methacrylate) (PMMA) block and a hydrophilic poly(*N*,*N*-2-(dimethylamino)-ethyl methacrylate) (PDMAEMA) block, (PMMA-b-PDMAEMA) were synthesized and blended with a poly(vinylidene fluoride) (PVDF) and GO solution followed by electrospun and coated with a hydrophilic polymer, poly(vinyl alcohol) (PVA). The prepared composite nanofibers exhibited super-hydrophilic properties and showed improved pure water flux for increasing the alkyl chain length and they also showed significant antibacterial activities against *E. coli* and *S. aureus*.

### 3.5. Functionalization with Antibiotics or Enzymes

Several antibiotics and antibacterial enzymes have been utilized against various bacterial pathogens [197,198]. Considering their evident toxicities via intravenous administration, the controlled and localized deployment is critical for achieving a secure and an enhanced effect at therapeutic concentrations. Graphene has been largely used as a promising drug delivery system due to its π- electron-rich surface and large specific surface area. Therefore, by combining the advantages of drug loading and inherent antibacterial activity, graphene could be functionalized with antibiotics and antibacterial enzymes for their extended delivery and enhanced antibacterial activity. In recent years, several graphene-based materials functionalized with antibiotics including vancomycin (Van), levofloxacin, cefalexin, ciprofloxacin and also enzymes such as lysozyme (Lys) have been explored and were found to provide the enhanced antibacterial capacity. 

**Figure 5:**
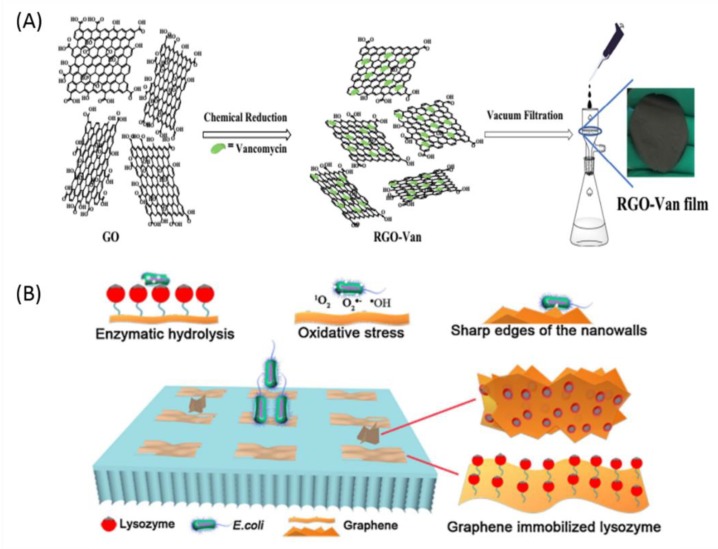
(**A**) Schematic representation for the fabrication of rGO-Van sheets and film. Reproduced from Reference [203], with permission from Elsevier, 2018. (**B**) Immobilization of lysozyme on graphene and illustration of the antibacterial mechanism of the hybrid membrane. Reproduced from Reference [86], with permission from Elsevier, 2015.

Van, a glycol-peptide antibiotic, binds to the cell wall of the Gram-positive bacteria via D-alanyl-D-alanine moieties [199,200] which could interfere in the transglycosylase step of the peptidoglycan biosynthesis. This results in lowering the cell wall rigidity and, thereby, causes bacterial cell death [201,202]. In addition, due to the π-π stacking interactions with graphene, Van could be rapidly released at the initial stage followed by sustained release, which could provide durable pathogen inhibition along with the inherent antibacterial activity of the graphene. Recently, Weng et al. [87] studied the controlled release of Van from the 3D porous self-assembled graphene-based composites for multiple benefits such as osteogenesis-promoting activity and antibacterial activities. Van was incorporated into the self-assembled 3D porous composites composed of nano-hydroxylapatite (nHA) and rGO. The antibacterial activity was evaluated against *S. aureus* and rabbit radius bone infected with *S. aureus* was used to determine activity of rGO-nHA composites for an infected bone defect treatment with the controlled release of Van. This graphene-based composite with Van provided the novel approach for the treatment of intractable orthopedic diseases. Most recently, Xu et al. [203] fabricated the Van-decorated rGO (rGO-Van) films. The GO was chemically reduced with Van in Tris HCl solution to produce rGO-Van suspension followed by vacuum-filtration through the nylon membrane and followed by drying to get the rGO-Van films (Figure 5A). Since glycosides in Van were involved in the reduction of GO, the remaining segments of Van still exhibited antibacterial activity towards the Gram-positive bacteria. rGO-Van films selectively inhibited *S. epidermidis* and *S. aureus* and also inhibited adhesion of the planktonic bacterial cells. Moreover, the rGO-Van films also exhibited the antibacterial activity in the rat infection model. The films healed the *S. aureus* infected wound and provided faster wound healing efficiency compared to GO film.

Huang et al. [89] tested the antibacterial performance of ciprofloxacin containing cross-linked GO-polyethyleneimine hybrid films. Due to cross-linking, the mechanical strength of the film was improved and showed slow release pattern of ciprofloxacin under various pH, which followed zero-order release kinetics. These ciprofloxacin loaded hybrid films exhibited good antibacterial activity. Xu et al. [88] covalently attached the cephalexin onto GO nanosheets via amide linkage to form cefalexin-grafted GO (GO-cefalexin) films with unique 2D layered structure and formed channels for release of cefalexin after immersing in water. GO-cefalexin films significantly inhibited the growth of *S. aureus* and *E. coli* as well as showed minimal cellular toxicity. Gao et al. [204] used functionalized GO modified polysebacic anhydride (PSA) composites for the controlled release of levofloxacin. PSA modified with a different feeding ratio of GO and exhibited much longer levofloxacin release behavior compared to pure PSA and the duration of levofloxacin release could be controlled by the molecular weight of GO modified PSA composites.

Enzymes have excellent catalytic properties under mild conditions such as specificity, selectivity, and activity [205] and produce safe and non-toxic products after catalysis, which confirms the green chemistry requirements [206]. One such enzyme, known as the lysozyme, catalyzes the bacterial cell wall hydrolysis and also acts as non-specific innate immunity moiety against the invasion of the pathogens [207]. However, lysozyme has inherently low durability and stability, which limits its applicability in the biomedical field. In recent years, the immobilization technique has been proven to be a successful tool for the improvement of enzyme properties [208]. Ding et al. [209] used silica nanotubes for the lysozyme immobilization and Saeki et al. [210] covalently immobilized the polyamide reverse-osmosis membranes with lysozyme. Recently, Duan et al. [86] studied the graphene immobilized lysozyme/polyethersulfone (PES) matrix membrane for its enhanced antibacterial mechanical and permeable properties. Lysozyme was immobilized on the surface of GO/rGO and blended in PES casting solution to prepare the PES ultra-filtration membrane. The water flux, mechanical strength, and hydrophilicity of hybrid membranes were significantly increased by immobilized lysozymes. Moreover, they also exhibited effective antibacterial activity against *E. coli*. In addition, GO immobilized lysozymes possessed higher antibacterial activity compared to rGO immobilized lysozymes (Figure 5B).

## 4. Multicomponent Composite Functionalization

Several graphene-based multicomponent composites have been prepared due to the strong Van der Waals force between the graphene sheets, which inhibits their function to improve their antibacterial applications [90,91,92,93,94,211,212]. Graphene-based multicomponent composites could be prepared by incorporating the metal, polymer, or NPs with graphene. NPs could improve properties of graphene due to their high surface area to volume ratio, which results in an appearance of valuable traits such as new chemical, electrical, mechanical, optical, and electro-optical properties that are different from their bulk properties. In addition, solubility of the graphene could be enhanced by incorporating polymers to form a stable dispersion. In particular, they improve adhesion to the substrate and exhibit synergistic antibacterial effects [213,214].

Liu et al. [215] used bacterial cellulose (BC) as the matrix for the synthesis of GO-TiO_2_ based multicomponent nanocomposites for photocatalytic antibacterial activity. The TiO_2_ NPs were densely anchored on the GO sheets to form GO-TiO_2_, which were filled into the porous BC matrix to obtain GO-TiO_2_/BC multicomponent nanocomposites. GO-TiO_2_/BC did not exhibit bacterial toxicity in the dark after incubation for a long time, whereas it produced the ROS and significantly inactivated the *S. aureus* after UV irradiation with the excellent photocatalytic antibacterial activity (91.3%). Xu et al. [216] prepared TiO_2_ NPs embedded self-assembled films composed of GO and CS for antibacterial and preservative properties. The films composed with the 1:20:4 ratio of GO, CS, and TiO_2_ NPs in the nanocomposite exhibited higher antibacterial activity against *B. subtilis* and *A. niger*. Moreover, these nanocomposite films did not exhibit any cytotoxicity against plant cells and mammalian somatic cells. Thus, because of their safety and selectivity, these nanocomposite films display great potential as antimicrobial coatings for the food preservation. Polyoxometalates (POM) are anionic clusters of metal oxides with remarkable physicochemical properties such as high thermal resistivity and water solubility [217] as well as interesting antibacterial properties [218,219]. Moghayedi et al. [220] fabricated rGo-phosphomolybdic acid (H_3_PMo_12_O_40_) (rGO-P-Mo) using the hydrothermal reduction method and evaluated the antibacterial activity against *E. coli* using colony counting and the micro-dilution method. rGO-P-Mo nanocomposites exhibited concentration-dependent activity and also exhibited higher antibacterial activity compared to pure P-Mo.

Teymourinia et al. [221] prepared the TiO_2_/Sb_2_S_3_/GQDs nanocomposites by using the solvothermal method to examine their antibacterial properties. TiO_2_/Sb_2_S_3_/GQDs nanocomposites exhibited the highest antibacterial activity compared to TiO_2_, Sb_2_S_3_, TiO_2_/GQDs, and Sb_2_S_3_/GQDs against *E. coli* and *S. aureus* with the MIC of 0.03 and 0.1, respectively. Calcium alginate (Ca-Alg) is a biodegradable, nontoxic, biocompatible, and renewable polysaccharide hydrogel possessing several industrial applications including biodegradable plastic packaging materials [222] and water treatment [223]. Martí et al. [224] fabricated the GO-Ca-Alg composite films to prevent *S. aureus* and methicillin-resistant *S. epidermidis* infections, which did not show toxicity for the human keratinocyte HaCaT cells. GO-Ca-Alg composite films were prepared using calcium chloride as a cross-linker and GO (0 to 1% *w*/*w*). The incorporation of 0.5% *w*/*w* GO content to the GO-Ca-Alg composite films provided very high antibacterial activity against both life-threatening pathogens. Hydroxyapatite (HAP) (Ca_10_(PO_4_)_6_(OH)_2_), which is a calcium phosphate bio-ceramics, has excellent biocompatibility, osteoconductivity, non-toxic, and osteoinduction and is utilized in various biomedical devices. Beiranvand et al. [225] prepared rGO/HAP/Ag multicomponent nanocomposites using the hydrothermal method and characterized them with various spectroscopic techniques. rGO/HAP/Ag nanocomposites reduced the toxic nitro-compounds into less toxic corresponding amines using NaBH_4_, which could be easily separated from the reaction mixture and re-used without any change in the structure because of their heterogeneous nature. Moreover, rGO/HAP/Ag nanocomposites exhibited better antibacterial activity against Gram-positive bacterial (*B. cereus* and *S. aureus*) than Gram-negative bacteria (*E. coli* and *Klebsiella pneumonia*). Faria et al. [226] functionalized the poly(lactide-co-glycolide)-chitosan (PLGA-CS) mats with GO-AgNPs via a chemical reaction between primary amine groups of PLGA-CS fibers and carboxyl groups of GO using 3-dimethylaminopropyl-N’-ethylcarbodiimide hydrochloride and N-hydroxysuccinimide. GO-AgNPs/PLGA-CS mats exhibited significant activity against both Gram-negative and Gram-positive bacteria.

**Figure 6:**
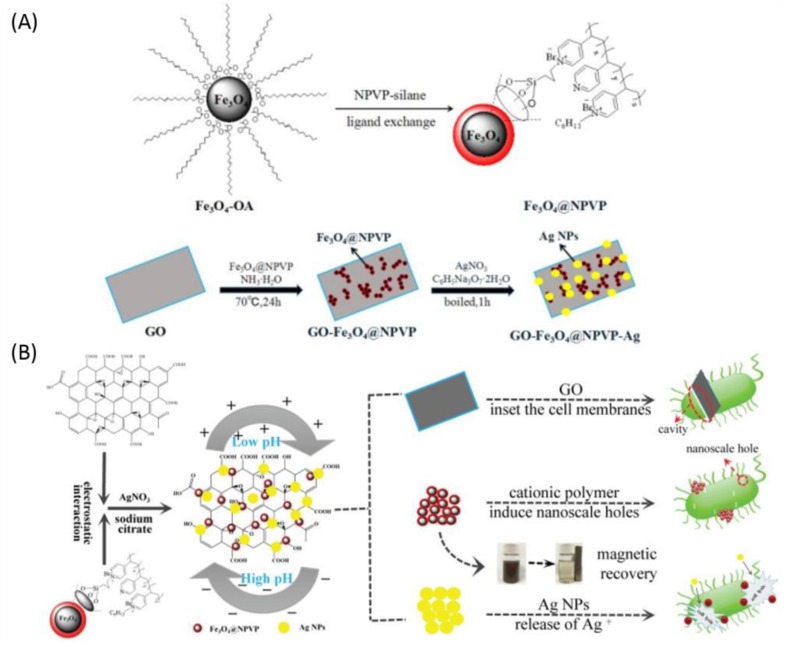
(**A**) Schematic representation for the preparation of GO-Fe_3_O_4_@NPVP-Ag. Reproduced from [228], with permission from Elsevier, 2018. (**B**) Antibacterial mechanism of action of GO-Fe_3_O_4_@NPVP-Ag against *E. coli*. Reproduced from Reference [228], with permission from Elsevier, 2018.

Zhang et al. [227] prepared the guanidyl functionalized graphene/polysulfone (GFG/PSF) matrix ultrafiltration antibacterial membranes using the non-solvent induced phase-separation method. The guanidyl functionalized graphene sheets were synthesized by amination. This was followed by guanidination, which exhibited excellent dispersibility in casting solution and compatibility with the matrix. The GFG/PSF mixed matrix membranes showed higher permeability and an anti-fouling property toward bovine serum albumin as well as remarkable antibacterial activity against *E. coli* and *S. aureus*. Li et al. [228] modified the Fe_3_O_4_ NPs surface with a cationic polymer, N-alkylated poly (4-vinylpyridine) (NPVP), and then combined it with GO sheets via electrostatic binding and, subsequently, deposited the AgNPs to form the multicomponent (GO-Fe_3_O_4_@NPVP-Ag) antibacterial nanocomposites. The nanocomposites exhibited excellent antibacterial activity against *E. coli* and *S. aureus* with MIC of 31.25 and 62.5 μg/mL, respectively, as well as low cytotoxicity on NIH-3T3 cells, which makes it a promising candidate as a bactericide in various antibacterial applications (Figure 6).

Noreen et al. [229] prepared visible light-sensitive GO-Ag-TiO_2_ nanocomposites using a hydrothermal process as a coating material for the control of the food-borne pathogen Campylobacter jejuni (*C. jejuni*). The nanocomposites effectively reduced the hydrophobicity, motility, auto-aggregation, and, ultimately, the growth of *C. jejuni* as well as inhibited the biofilm formation. Moreover, no significant cytotoxicity was observed toward human cell lines. Li et al. [230] fabricated chitosan chloride-GO (CSCl@GO) composites modified quartz sand (CSCl@GO/QS) filter media in order to control the bacterial pollution form of the water treatment. CSCl@GO composites were prepared using a solution blending method and investigated for the antibacterial mechanism using *E. coli* and *S. aureus*, which were completely inactivated after CSCl@GO (100 mg/L) composites treatment for 15 min. The antibacterial efficiency of CSCl@GO composites reached 95.74% in circulating cooling water with a GO mass fraction of 0.6%. The antibacterial efficiency of CSCl@GO/QS filter media was above 90% even after backwashing three times, which suggests that nanocomposites can be applied as a promising antibacterial and anti-biofilm coating material to control the spreading of *C. jejuni*.

## 5. Biosafety

The increasing applications and production of graphene-based materials have enhanced the risk of their unintentional exposure. However, there are conflicting results on their biosafety and biocompatibility partially because of the large variation in their physicochemical properties. To enlighten the biosafety related to graphene-based antibacterial materials, it is imperative to discuss the interactions between graphene and bio-macromolecules such as proteins and nucleic acids. The graphene well interacts with the deoxyribose nucleic acid (DNA) and its binding affinity with single-stranded DNA via noncovalent interactions such as hydrophobic forces and π-π stacking interactions is much higher than with double-stranded DNA or tertiary DNA structures. Similarly, since proteins have physiological activities within the cells in all living organisms, recognition of their interactions with graphene is necessary to study the biological toxicity and cellular uptake of graphene. Jung et al. [231] found that the antibody particularly links on the carboxylic acid rich edges and folded structures of graphene. Gan et al. [232] studied the interactions between graphene and blood proteins and revealed that they bound via π-π interactions. Furthermore, Tan et al. [233] found PEGylated graphene generated nano-interface, which reduced serum protein binding and complemented C3 activation. All these findings proved the existence of interactions between graphene and several proteins/nucleic acids.

In recent years, there are several studies describing the potentially toxic effects of the graphene-based materials in human cells and animals. To investigate the biocompatibility of the GO, Wang et al. [234] studied the effect of GO on human fibroblast cells and mice. GO did not exhibit any cytotoxicity to human fibroblast cells with less than a 20 μg/mL dose whereas it exhibited cytotoxicity with doses of more than 50 μg/mL, such as inducing cell apoptosis, decreasing cell adhesion, and entering into lysosomes, endoplasm, mitochondrion, and the nucleus of the cells. However, in case of mice, GO did not exhibit toxicity with low (0.1 mg) and middle (0.25 mg) doses but exhibited chronic toxicity with a high (0.4 mg) dose, such as 4/9 mice deaths and granuloma formation in the lung, spleen, kidney, and liver, which could not be cleared through the kidney. Liao et al. [235] reported the cytotoxicity related to graphene and GO in human erythrocytes and skin fibroblasts and demonstrated that the particulate state, particle size, and surface charge/oxygen content of the graphene had a strong impact on the toxicological and biological responses to the red blood cells. The cytotoxicity of graphene sheets and GO was investigated using trypan blue exclusion, water-soluble tetrazolium salt, and the ROS assay, which revealed that compacted graphene sheets damaged the mammalian fibroblasts when compared to less densely packed GO. Therefore, it suggests that the toxicity of graphene sheets and GO depends on the mode of interaction with the cells (adherent or suspension cell) and exposure environment (whether aggregation occurs or not). Hu et al. [26] investigated the cytotoxicity of GO using protein corona mediated mitigation and demonstrated that the direct interaction between GO and the cell membrane resulting in physical damage to the cell membrane was the main cause of cytotoxicity. In another study, Sasidharan et al. [236] found that pristine graphene accumulated in the cell membrane, which leads to apoptosis due to the high oxidative stress whereas its carboxyl functionalized derivative internalized in the cells without any cytotoxicity. In conclusion, most of the studies considered the material concentration and contact time as the parameter for the effect of toxicity and increasing these variables decreased the cell viability.

**Figure 7:**
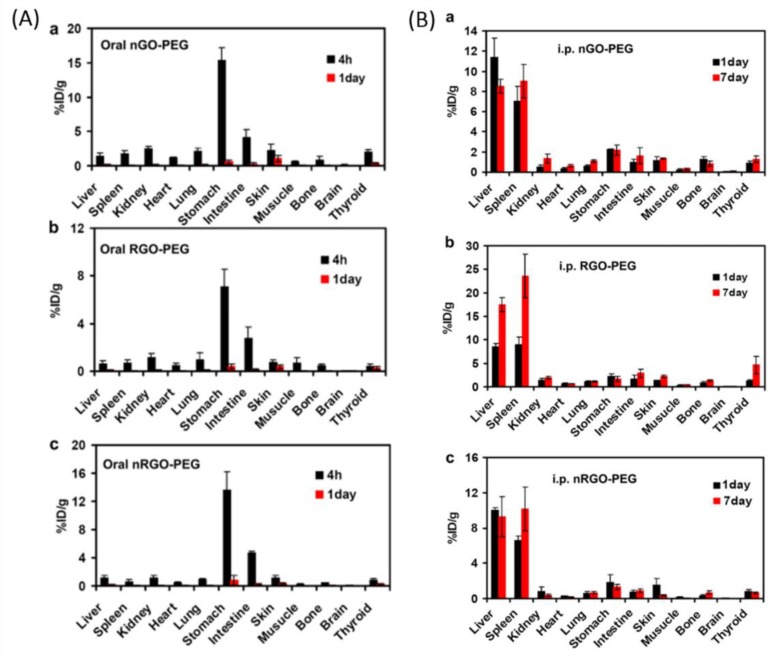
Biodistribution of (a) nGO-PEG, (b) RGO-PEG, and (c) nRGO-PEG after (**A**) oral administration and (**B**) intraperitoneal (i.p.) administration. Reproduced from Reference [238], with permission from Elsevier, 2013.

Furthermore, lateral sizes of the graphene-based materials also have an impact on biodistribution and cellular uptake. In this regard, Ma et al. [237] studied the role of the lateral size of GO in activating the macrophages and stimulating pro-inflammatory responses in the cells and animals and found that, in comparison to the smaller GO, a larger counterpart showed stronger adsorption onto the plasma membrane, which elicited more potent activation of the NF-κB pathways. On the other hand, smaller GO sheets were easily ingested inside the cells. Therefore, it was suggested that larger GO sheets promoted the M1 polymerization, which was associated with an increased inflammatory cytokine production and recruitment of the immune cells. To understand the fate of the GO and their derivatives in the animals after intraperitoneal injection or oral feeding, Yang et al. [238] systematically investigated the potential toxicity and in vivo biodistribution of GO sheets and PEG-functionalized GO sheets with different surface coating and sizes. No tissue uptake was observed for PEG-functionalized GO sheets via oral administration (Figure 7A). However, high accumulation of PEG-functionalized GO sheets, but not of GO, was observed in the reticuloendothelial (RES) system (including spleen and liver) after intraperitoneal injection (i.p.) (Figure 7B). Further hematological analysis and histological examination of the organ slices revealed that even GO and PEG-functionalized GO sheets would be retained in the mouse body for a long time after intraperitoneal injection, their toxicity was insignificant.

In order to speed up the clinical or commercial use of graphene-based materials, it is important to evaluate the biosafety of graphene-based materials in mammals. Most existing studies reported that the mammal’s cell viability decreased after exposure to graphene-based materials. There are several issues related to graphene toxicology, which are needed to be taken into account such as toxicity criteria, the valid amount, and joint toxicity. The acceptance criterion for the toxicity tests such as statistical procedures, choice of statistical assumptions, or experimental conditions is needed in order to compare toxicity obtained with different conditions. In addition, a certain valid concentration for the toxicity should be defined in order to get the correct and acceptable results related to the cytotoxic effects. Moreover, a large difference between joint toxicity and single toxicity have been reported in literature [239,240], which might be due to the synergetic effect between graphene with other materials. Therefore, joint toxicity related concerns also need more attention.

## 6. Concluding Remarks and Future Perspective

The global summary of recent advances on the graphene-based antibacterial materials has been enlightened in this review. Inherent antibacterial activity of the graphene-family has been discussed, which fueled up further research on graphene-based materials. Moreover, graphene has been utilized via functionalization with various nanomaterials such as metal ion/oxide NPs, polymers, enzymes, and photocatalytic materials leading to enhanced antibacterial activity. Recently, graphene has also been used as a carrier for controlled release of conventional antibiotics with improved therapeutic efficacy and reduced toxicity. Furthermore, various multicomponent materials have been developed by using graphene and different nanomaterials, which provided the higher antibacterial activity due to a synergetic effect. Overall, recent research advances in the field of fabrication of various novel graphene-based materials, their interaction with biomolecules, cytotoxicity, in vivo toxicity, and their applications in antibacterial activities, water purification, drug delivery, wound healing, and coating materials.

Despite several achievements, there are still some difficulties and challenges associated with graphene-based materials. The main challenge is understanding the detailed information about the interaction between bacteria and graphene-based materials. The contribution of each physicochemical properties such as basal planes, lateral size, or oxygen content is still not clear in terms of the antibacterial activity of graphene-based materials. Therefore, meaningful efforts are needed to understand the factors affecting the interactions and mechanism involved in induced bacterial death. Secondly, since graphene-based materials are used in various applications, the toxicity associated with them has diverted great attention among the researchers toward public health and environmental risks. Despite a large number of studies on their toxicity, their effects on normal mammalian cells and the environment during practical use are still being debated. This might be due to the inconsistencies in the results and lack of universal acceptance criteria for the toxicity of the graphene-based materials, which urgently need to be sorted out before applying them in practice. Thirdly, the antibacterial tests on the majority of graphene-based materials were applied mainly against *E. coli* and *S. aureus*. Therefore, it is very important to investigate the materials with other pathogenic species to demonstrate a broad bactericidal range and keep the account of increasing antibiotic resistance among various bacteria and their association as a serious threat to worldwide public health.

Despite certain challenges, the antibacterial materials have become a necessary tool in modern medical science and several surgical operations cannot be executed without using them. Therefore, the urgent demand for developing new and improved antibacterial therapies to fight against infections has become crucial. It is hoped that the advances that have been made by the researchers in terms of understanding and developing graphene-based materials will prove fruitful in the near future. We prospect that the information provided in this review about the recent advances on the graphene-based materials such as the antibacterial agents will help scientists understand the ongoing developments, will excite novel ideas to conquer the associated challenges, and will assist the fabrication of new and improved antibacterial materials.

## Figures and Tables

**Table 1:** Bactericidal characters of graphene-based materials.

Graphene Materials	Bacteria Model	Evaluation Method	Concentration	Inhibition	Reference
**Graphene family**					
**GO**	*S. aureus*/*P. aeruginosa*	ADA	300 µg/mL	93.7/48%	[68]
**GO**	*P. aeruginosa*	Plate count	175 µg/mL	100%	[65]
**rGO**	*E. coli*	Plate count	100 µg/mL	88%	[66]
**rGO**	*P. aeruginosa*	Plate count	175 µg/mL	100%	[65]
**Functionalized with Silver NPs**					
**GO-AgNPs**	*E. coli*/*S. aureus*	Plate count	10 µg/mL	100%	[69]
**GO-Ag_3_PO_4_ NPs**	*E. coli*/*S. aureus*	Plate count	300 µg/mL	92.8/100%	[70]
**rGO-AgNPs**	*E. coli*	Plate count	40 µg/mL	100%	[71]
**rGO-Ag/Ag_2_S**	*E. coli*	Plate count	N/A	97.76%	[72]
**Photocatalytic Functionalization**					
**rGO-TiO_2_**	*E. coli*/*S. Aureus*	ADA	N/A	N/A	[73]
**rGO-ZnO**	*E. coli*	Plate count	3 × 10^3^ µg/mL	100%	[74]
**GO-ZnO**	*E. coli*	Plate count	500 µg/mL	100%	[75]
**GO-CdS**	*E. coli/B. subtilis*	Plate count	N/A	100%	[76]
**Functionalization with Other Metal Ions/Oxides**					
**rGO-Cu_2_O**	*E. coli*/*S. aureus*	Plate count	40 µg/mL	70/65%	[77]
**GO-Fe_3_O_4_**	*E. coli*	Plate count	300 µg/mL	91.5%	[78]
**GO–Fe_2_O_3_**	*E. coli*	Plate count	100 µg/mL	97%	[79]
**GO–MnFe_2_O_4_**	*E. coli*	Plate count	100 µg/mL	82%	[80]
**GO-Bi_2_WO_6_**	Mixed culture	Plate count	250 µg/mL	100%	[81]
**Functionalization with Polymers**					
**PVA-CS-GO**	*E. coli*/*B. subtillis*	ADA	N/A	1.25/1.40 mm	[82]
**PDMS-GO-DMA**	*E. coli*/*S. aureus*	Plate count	N/A	~40%	[83]
**GO-CS**	*E. coli*/*S. aureus*	ADA	2 wt %	N/A	[84]
**GO-PEG-PHGC**	*E. coli*/*S. aureus*	Plate count	4 × 10^3^ µg/mL	N/A	[85]
**Functionalized with Antibiotics or Enzymes**					
**GO-Lys**	*E. coli*	Plate count	32–512 μg/mL	68%	[86]
**rGO-Van-nHA**	*S. aureus*	KBA	1–4% Van	N/A	[87]
**GO-cefalexin**	*E. coli*/*S. aureus*	ADA	N/A	6.3/6.9 mm	[88]
**GO-PEI-ciprofloxacin**	*E. coli*	ADA	1 cm^2^ (film)	100%	[89]
**Multicomponent Composite Functionalization**					
**GO-Ag NPs-PAA**	*E. coli*/*S. aureus*	ADA	N/A	9.9/11.4 mm	[90]
**GO-Ag NPs-PDA**	*E. coli*	ADA	25 µg/mL	23.7 mm	[91]
**rGO-Ag NPs-PDDA**	*E. coli*	Plate count	50 µg/mL	100%	[92]
**rGO-PEI-AgNPs-Fe_2_O_3_**	*E. coli*	Plate count	0.1 µg/mL	99.9%	[93]
**rGO-Ag-CoFe_2_O_4_**	*E. coli*/*S. aureus*	Plate count	12.2 µg/mL	97–99%	[94]

ADA: agar diffusion assay. CS: chitosan. DMA: dopamine methacrylamide. GO: graphene oxide. Lys: lysozyme. N/A: not applicable. nHA: nano-hydroxylapatite. PAA: poly(acrylic acid). PDA: polydopamine. PDDA: poly (diallyl dimethyl ammonium chloride). PDMS: poly(dimethylsiloxane). PEG: poly (ethylene glycol). PHGC: polyhexamethylene guanidine hydrochloride. PEI: polyethylenimine. PVA: polyvinyl alcohol. Ag: Silver. NPs: Nanoparticles. rGO: reduced graphene oxide. Van: Vancomycin.
